# Supplementary material for: Systematic Review: Strategies for Improving HIV Testing and Detection Rates in European Hospitals
Source: Microorganisms. 2024 Jan 25;12(2):254. doi: 10.3390/microorganisms12020254 (PMC10892502; doi:10.3390/microorganisms12020254)
Supplement: Supplementary file 1 [file microorganisms-12-00254-s001.zip › microorganisms-2814635-supplementary/S1 PICO.pdf]

Supplement 1 (S1): PICO framework - HIV testing strategies in European hospitals

**Criteria**	**Inclusion criteria**	**Exclusion criteria**
Population	Studies conducted in European hospitals<br>* adults aged 18 year or above<br>* in the hospital, outpatient department of a hospital or emergency care department of a hospital<br>* in the in- or outpatient setting<br>* in the WHO European region	* adults already diagnosed with HIV (regardless of treatment)<br>* individuals who already tested HIV negative in the last 6 months<br>*studies conducted in the primary care unit, dedicated STI, tuberculosis or antenatal care clinic or community services<br>* health centre or health care facility
Intervention	Hospital based strategies or interventions aimed at improving the detection of occult HIV infections.<br><br>These strategies may include, but are not limited to,<br>a. changes in testing protocols or algorithms, including<br>* symptom-based approaches<br>* implementing opt-out strategy<br>* enhanced testing of high-risk populations<br>* electronic reminders<br>b. use of advanced testing technologies,<br>c. educational programs for healthcare providers,<br>d. implementation plan for guidelines for HIV testing and diagnosis	Studies that do not investigate strategies or interventions related to the detection of occult HIV infections.<br><br>Studies reporting on interventions outside the hospital setting like community-based testing, online interventions, self-testing or peer-led initiatives<br><br>Studies that report HIV testing rates or HIV prevalence numbers, without the intention to implement or study a strategy to improve this.
Outcome	Studies reporting quantitative data on the effectiveness of the strategy, preferably a change in HIV testing rate or detection of HIV infections.<br><br>Primary:<br>* HIV case finding<br>(HIV cases/ population of interest)<br><br>Secondary:<br>* improvement of HIV testing rate<br>(HIV tests/ population of interest)	Studies not reporting on impact on HIV testing rate or case finding
Study	* No publication date restriction<br>* No language restriction<br>* Any study design, including:	* Reviews, posters, opinion pieces, conference abstracts, news briefing, qualitative studies.

	Peer-reviewed primary research studies, randomised controlled trials (RCTs), non-randomized quasi-experimental studies, observational studies including cohort studies and cross-sectional studies, case-control studies	* Non-peer-reviewed articles
