# Supplementary material for: Systematic Review: Strategies for Improving HIV Testing and Detection Rates in European Hospitals
Source: Microorganisms. 2024 Jan 25;12(2):254. doi: 10.3390/microorganisms12020254 (PMC10892502; doi:10.3390/microorganisms12020254)
Supplement: Supplementary file 1 [file microorganisms-12-00254-s001.zip › microorganisms-2814635-supplementary/S2 Full search.pdf]

Supplement 2 (S2): Full search - HIV testing strategies in European hospitals
Search strings and records found (4-10-2023)

**Database searched**	**Platform**	**Years of coverage**	**Records**	**Records after duplicates removed**
Medline ALL	Ovid	1946 - Present	1,347	1,344
Embase	Embase.com	1971 - Present	2,356	1,274
Web of Science Core Collection*	Web of Knowledge	1975 - Present	895	84
**Total**			**4,598**	**2,702**

*Science Citation Index Expanded (1975-present) ; Social Sciences Citation Index (1975-present) ; Arts & Humanities Citation Index (1975-present) ; Conference Proceedings Citation Index- Science (1990-present) ; Conference Proceedings Citation Index- Social Science & Humanities (1990-present) ; Emerging Sources Citation Index (2005-present)

No other database limits were used than those specified in the search strategies.

Medine 1347

(HIV Testing/ OR ((HIV Infections/ OR HIV/) AND (Mass Screening/ OR Diagnostic Tests, Routine/)) OR ((HIV OR human-immunodeficien*-virus* OR human-immun*-deficien*-virus*) ADJ3 (test* OR clia OR elisa OR screen*)).ab,ti,kw.) AND (exp Hospitals/ OR Inpatients/ OR Hospitalization/ OR exp Hospital Departments/ OR (hospital* OR clinic OR clinics OR ward* OR ((emergenc* OR intensive-care* OR critical-care*) ADJ3 (unit* OR department*))).ab,ti,kw.) AND (exp Europe/ OR Israel/ OR Tajikistan/ OR Turkmenistan/ OR Uzbekistan/ OR Kazakhstan/ OR Kyrgyzstan/ OR (Europ* OR Albania* OR Andorr* OR Armenia* OR Austria* OR Azerbaija* OR Balkan* OR Baltic* OR Belarus* OR Belgi* OR Benelux* OR Bosnia* OR Herzegovina* OR Bulgaria* OR Channel-Island* OR Croatia* OR Czech* OR Denmark* OR Danish* OR England* OR Estonia* OR Faroe* OR Finland* OR Finnish* OR France* OR French* OR Georgia* OR German* OR Gibraltar* OR Great-Brit* OR British* OR Greece* OR Greek* OR Greenland* OR Hungar* OR Iceland* OR Ireland* OR Irish* OR Israel* OR Isle-of-Man* OR Manx* OR Ital* OR Kazakh* OR Kyrgyzstan* OR Kosov* OR Latvia* OR Liechtenstein* OR Lithuania* OR Luxembourg* OR Malt* OR Moldova* OR Monaco* OR Montenegr* OR Netherland* OR Dutch* OR Norway* OR Norwegian* OR Poland* OR Polish* OR Portug* OR Macedoni* OR Romania* OR Russia* OR San-Marin* OR Scandinavia* OR Scotland* OR Scottish* OR Serbia* OR Slovak* OR Slovenia* OR Spain* OR Spanish* OR Sweden* OR Swedish* OR Switzerland* OR Swiss* OR Tajikistan* OR Turkmen* OR Ukrain* OR United-Kingdom* OR Uzbeki* OR Vatican* OR Wales* OR Welsh* OR uk OR london OR barcelona OR madrid).ab,ti,kw.) NOT ((exp child/ OR exp infant/ OR pediatrics/ OR adolescent/) NOT exp adult/) NOT (exp animals/ NOT humans/)

Embase 2356

('HIV test'/exp OR (('Human immunodeficiency virus infection'/de OR 'Human immunodeficiency virus'/de) AND (screening/de OR 'screening test'/de OR 'mass screening'/de OR 'diagnostic test'/de)) OR ((HIV OR human-immunodeficien*-virus* OR human-immun*-deficien*-virus*) NEAR/3 (test* OR clia OR elisa OR screen*)):ab,ti,kw) AND (hospital/exp OR 'hospital patient'/exp OR hospitalization/de OR 'hospital admission'/de OR ward/exp OR (hospital* OR clinic OR clinics OR ward* OR ((emergenc* OR intensive-care* OR critical-care*) NEAR/3 (unit* OR department*))):ab,ti,kw) AND (Europe/exp OR Israel/de OR Tajikistan/de OR Turkmenistan/de OR Uzbekistan/de OR Kazakhstan/de OR Kyrgyzstan/de OR (Europ* OR Albania* OR Andorr* OR Armenia* OR Austria* OR Azerbaija* OR Balkan* OR Baltic* OR Belarus* OR Belgi* OR Benelux* OR Bosnia* OR Herzegovina* OR Bulgaria* OR Channel-Island* OR Croatia* OR Czech* OR Denmark* OR Danish* OR England* OR Estonia* OR Faroe* OR Finland* OR Finnish* OR France* OR French* OR Georgia* OR German* OR Gibraltar* OR Great-Brit* OR British* OR Greece* OR Greek* OR Greenland* OR Hungar* OR Iceland* OR Ireland* OR Irish* OR Israel* OR Isle-of-Man* OR Manx* OR Ital* OR Kazakh* OR Kyrgyzstan* OR Kosov* OR Latvia* OR Liechtenstein* OR Lithuania* OR Luxembourg* OR Malt* OR Moldova* OR Monaco* OR Montenegr* OR Netherland* OR Dutch* OR Norway* OR Norwegian* OR Poland* OR Polish* OR Portug* OR Macedoni* OR Romania* OR Russia* OR San-Marin* OR Scandinavia* OR Scotland* OR Scottish* OR Serbia* OR Slovak* OR Slovenia* OR Spain* OR Spanish* OR Sweden* OR Swedish* OR Switzerland* OR Swiss* OR Tajikistan* OR Turkmen* OR Ukrain* OR United-Kingdom* OR Uzbeki* OR Vatican* OR Wales* OR Welsh* OR uk OR london OR barcelona OR madrid):ab,ti,kw) NOT (juvenile/exp NOT adult/exp) NOT ([conference abstract]/lim AND [2000-2021]/pd) NOT ([animals]/lim NOT [humans]/lim)

Web of science 895

TS=((((HIV OR human-immunodeficien*-virus* OR human-immun*-deficien*-virus*) NEAR/2 (test* OR clia OR elisa OR screen*))) AND ((hospital* OR clinic OR clinics OR ward* OR ((emergenc* OR intensive-care* OR critical-care*) NEAR/2 (unit* OR department*)))) AND ((Europ* OR Albania* OR Andorr* OR Armenia* OR Austria* OR Azerbaija* OR Balkan* OR Baltic* OR Belarus* OR Belgi* OR Benelux* OR Bosnia* OR Herzegovina* OR Bulgaria* OR Channel-Island* OR Croatia* OR Czech* OR Denmark* OR Danish* OR England* OR Estonia* OR Faroe* OR Finland* OR Finnish* OR France* OR French* OR Georgia* OR German* OR Gibraltar* OR Great-Brit* OR British* OR Greece* OR Greek* OR Greenland* OR Hungar* OR Iceland* OR Ireland* OR Irish* OR Israel* OR Isle-of-Man* OR Manx* OR Ital* OR Kazakh* OR Kyrgyzstan* OR Kosov* OR Latvia* OR Liechtenstein* OR Lithuania* OR Luxembourg* OR Malt* OR Moldova* OR Monaco* OR Montenegr* OR Netherland* OR Dutch* OR Norway* OR Norwegian* OR Poland* OR Polish* OR Portug* OR Macedoni* OR Romania* OR Russia* OR San-Marin* OR Scandinavia* OR Scotland* OR Scottish* OR Serbia* OR Slovak* OR Slovenia* OR Spain* OR Spanish* OR Sweden* OR Swedish* OR Switzerland* OR Swiss* OR Tajikistan* OR Turkmen* OR Ukrain* OR United-Kingdom* OR Uzbeki* OR Vatican* OR Wales* OR Welsh* OR uk OR london OR barcelona OR madrid))) AND DT=(article) AND LA=(english)
