# Supplementary material for: Systematic Review: Strategies for Improving HIV Testing and Detection Rates in European Hospitals
Source: Microorganisms. 2024 Jan 25;12(2):254. doi: 10.3390/microorganisms12020254 (PMC10892502; doi:10.3390/microorganisms12020254)
Supplement: Supplementary file 1 [file microorganisms-12-00254-s001.zip › microorganisms-2814635-supplementary/S3 PRISMA checklist.pdf]

Supplement 3 (S3): PRISMA 2020 Checklist – HIV testing strategies in European hospitals

Section and Topic	Item #	Checklist item	Location where item is reported
TITLE			
Title	1	In a review examining what proactive strategies are used to improve the HIV testing and detection rate in European hospitals, and what is the evidence for their effectiveness, the authors identify the report as systematic review	Title page
ABSTRACT			
Abstract	2	In a review examining what proactive strategies are used to improve the HIV testing and detection rate in European hospitals, and what is the evidence for their effectiveness , the authors summarise the objectives, eligibility criteria, databases searched, and methods to assess to evaluate the key interventions in order to actively increase HIV testing rates and case detection in European hospitals.	Abstract
INTRODUCTION			
Rationale	3	In a review examining what proactive strategies are used to improve the HIV testing and detection rate in European hospitals, and what is the evidence for their effectiveness, the authors report the knowledge gap and limitations of the existing evidence base.	Background
Objectives	4	In a review examining what proactive strategies are used to improve the HIV testing and detection rate in European hospitals, and what is the evidence for their effectiveness authors report a following objective of the review: to identify the strategies employed in European hospitals to enhance HIV testing rates and the detection of HIV cases, as well as to evaluate their efficacy.	Background
METHODS			
Eligibility criteria	5	In a review examining what proactive strategies are used to improve the HIV testing and detection rate in European hospitals, and what is the evidence for their effectiveness the authors report the types of studies, participants, interventions, comparators, and outcomes that were eligible for inclusion in the review. Predefined inclusion and exclusion criteria were established based on the Population, Intervention, Comparison, Outcome (PICO) framework, outlined in appendix 1.	Methods, selection criteria
Information sources	6	In a review examining what proactive strategies are used to improve the HIV testing and detection rate in European hospitals, and what is the evidence for their effectiveness the authors list the electronic bibliographic databases (with dates of coverage for each), and websites searched. All study types published after peer-review in journals were considered for inclusion, excluding reviews, posters, and conference abstracts.They also indicate that reference lists of all eligible study reports were reviewed and forward citation tracking of all eligible study reports was conducted.	Methods, selection criteria
Search strategy	7	The detailed search, available in appendix 2, employed key terms such as 'HIV testing' or 'HIV infections' coupled with terms indicating hospitals and the fifty-three individual countries included in the WHO European region, as published by the WHO.	Methods, search strategy
Selection process	8	The screening process, facilitated by Rayyan, followed a two-step approach, involving an initial assessment of titles and abstracts, followed by a full-text evaluation of selected eligible articles. Discrepancies in article selection between the two reviewers (K.V. and M.V.) were resolved through discussion with a third reviewer (C.R.) consulted in the second round until consensus.	Methods, search startegy
Data collection process	9	Two reviewers (K.V. and M.V.) extracted data from the selected studies using a standardized data extraction table in Excel. Inter-reviewer validation was implemented by cross-validation of the extracted data.	Methods, data extraction
Data items	10a	Inter-reviewer validation was implemented by cross-validation of the extracted data.	Methods, data extractions
	10b	In this review examining what proactive strategies are used to improve the HIV testing and detection rate in European hospitals, and what is the evidence for their effectiveness the authors list and define variables for which data were sought, including characteristics of the report, participants, study design and intervention:	Methods, data extraction
Study risk of bias assessment	11	Quality assessment of the non-randomized studies was performed using the Risk of Bias in Non-randomized Studies of Interventions (ROBINS-I) tool.Two independent reviewers (K.V. and M.V.) evaluated each study, assessing seven domains. To ensure the accuracy and reliability of the quality assessment, a cross-checking process was conducted by two additional reviewers (C.J. and O.S.). Discrepancies identified during this phase were resolved through discussion with the first author.	Methods, quality assessment

Supplement 3 (S3): PRISMA 2020 Checklist – HIV testing strategies in European hospitals

Section and Topic	Item #	Checklist item	Location where item is reported
Effect measures	12	HIV test rate (defined as the number of tests performed as a proportion of the eligible population)<br>HIV positivity rate (defined as the new HIV diagnoses as a proportion of the tested population)<br>HIV case finding (defined as newly diagnosed HIV infections as a proportion to the studied population)	NA
Synthesis methods	13a	We categorized the data by the main interventions studied, and we considered a testing-all strategy the primary intervention if combined with other interventions because of the profound impact on the number of eligible persons.	NA
	13b	NA	NA
	13c	NA	NA
	13d	NA	NA
	13e	NA	NA
	13f	NA	NA
Reporting bias assessment	14	In a review examining what proactive strategies are used to improve the HIV testing and detection rate in European hospitals, and what is the evidence for their effectiveness the authors report publication bias as studies as non-peer-reviewed publications, such as conference abstracts, were excluded.In addition see item 11	Data extraction
Certainty assessment	15	NA	NA
RESULTS			
Study selection	16a	The initial search yielded in total 4,598 articles, resulting in 2,702 records after duplicates were removed (Figure 1). Following the screening of titles and abstracts, 103 articles were selected for full-text review. Two studies were added by reference checking, making a total of 29	Results
	16b	After application of inclusion and exclusion criteria, and exclusion of a duplicate publication, 27 studies were included in the qualitative synthesis. Two studies were added by reference checking, making a total of 29	Results
Study characteristics	17	In a review examining review examining what proactive strategies are used to improve the HIV testing and detection rate in European hospitals, and what is the evidence for their effectiveness the authors include a table presenting for each included study the citation, study design, country, sample size, settings, exposure details and outcomes assessed.	Results
Risk of bias in studies	18	In a review the authors present a table indicating the domain-specific and overall risk of bias judgement for each study result, and include justification for assessments.	Results, Quality assessment
Results of individual studies	19	NA	NA
Results of syntheses	20a	NA	NA
	20b	NA	NA
	20c	NA	NA
	20d	NA	NA
Reporting biases	21	The results of the quality assessment revealed variations in the risk of bias across the studies	Table 4
Certainty of evidence	22	In a review the authors report their certainty in the abstract text and present tables including certainty judgements for several outcomes.	Table 1,Table 2, Table 3, Table 4
DISCUSSION			
Discussion	23a	In a review, the authors describe how their review differs to the methods used in previous reviews.	Discussion

Supplement 3 (S3): PRISMA 2020 Checklist – HIV testing strategies in European hospitals

Section and Topic	Item #	Checklist item	Location where item is reported
	23b	In a review the authors describe several limitations of the included studies	Discussion
	23c	In a review the authors report several limitations of the review processes used.	Discussion
	23d	In a review the authors list implications for future research.	Discussion, Conclusion
OTHER INFORMATION			
Registration and protocol	24a	NA (Registration in PROSPERO under number 47228 was intended but not approved, due to the short turnaround time from initiation to submission (3 months). Additionally, the progress was too rapid to meet the formal requirements."	NA
	24b	NA	NA
	24c	NA	N/A
Support	25	Sources of financial or non-financial support for the review are described	Funding
Competing interests	26	Competing interests of the authors are declared in the section ‘conflict of interest’	Conflict of Interest
Availability of data, code and other materials	27	Data collection forms and other materials are available upon request	Upon request

From: Page MJ, McKenzie JE, Bossuyt PM, Boutron I, Hoffmann TC, Mulrow CD, et al. The PRISMA 2020 statement: an updated guideline for reporting systematic reviews. BMJ 2021;372:n71. doi: 10.1136/bmj.n71 (http://www.prisma-statement.org/)
