# Supplementary material for: Systematic Review: Strategies for Improving HIV Testing and Detection Rates in European Hospitals
Source: Microorganisms. 2024 Jan 25;12(2):254. doi: 10.3390/microorganisms12020254 (PMC10892502; doi:10.3390/microorganisms12020254)
Supplement: Supplementary file 1 [file microorganisms-12-00254-s001.zip › microorganisms-2814635-supplementary/S4 RCT risk of bias tool.pdf]

Supplement 4 (S4): Quality assessment of RCT based on Revised Cochrane risk-of-bias tool for randomized trials (RoB 2) – HIV testing strategies within European hospitals

Quality assessment of:

1. Gillet C, Darling KEA, Senn N, Cavassini M, Hugli O. Targeted versus non-targeted HIV testing offered via electronic questionnaire in a Swiss emergency department: A randomized controlled study. PLoS One. 2018;13(3):e0190767.
2. Leblanc J, Hejblum G, Costagliola D, Durand-Zaleski I, Lert F, de Truchis P, et al. Targeted HIV Screening in Eight Emergency Departments: The DICI-VIH Cluster-Randomized Two-Period Crossover Trial. Ann Emerg Med. 2018;72(1):41-53 e9.

Tool: Revised Cochrane risk-of-bias tool for randomized trials (RoB 2). Edited by Julian PT Higgins, Jelena Savović, Matthew J Page, Jonathan AC Sterne on behalf of the RoB2 Development Group. Version of 22 August 2019. Available from: https://methods.cochrane.org/bias/resources/rob-2-revised-cochrane-risk-bias-tool-randomized-trials

Study details

Reference: Targeted versus non-targeted HIV testing offered via electronic questionnaire in a Swiss emergency department: A randomized controlled study(1)

Study design

X Individually-randomized parallel-group trial

☐ Cluster-randomized parallel-group trial

☐ Individually randomized cross-over (or other matched) trial

For the purposes of this assessment, the interventions being compared are defined as

Experimental: non-targeted HIV screening

Comparator: targeted testing using FOPH testing criteria

Specify which outcome is being assessed for risk of bias

to determine whether non-targeted screening was superior to targeted testing using FOPH testing criteria in terms of the number of free rapid HIV tests performed among eligible patients during their ED visit.

Specify the numerical result being assessed. In case of multiple alternative analyses being presented, specify the numeric result (e.g. RR = 1.52 (95% CI 0.83 to 2.77) and/or a reference (e.g. to a table, figure or paragraph) that uniquely defines the result being assessed.

Test acceptance and primary/final HIV testing rate (in percentage)

Is the review team's aim for this result…?

x to assess the effect of *assignment to intervention* (the 'intention-to-treat' effect)

☐ to assess the effect of *adhering to intervention* (the 'per-protocol' effect)

If the aim is to assess the effect of *adhering to intervention*, select the deviations from intended intervention that should be addressed (at least one must be checked):

X occurrence of non-protocol interventions
☐ failures in implementing the intervention that could have affected the outcome
☐ non-adherence to their assigned intervention by trial participants

Which of the following sources were <u>obtained</u> to help inform the risk-of-bias assessment? (tick as many as apply)

X Journal article(s) with results of the trial
☐ Trial protocol
☐ Statistical analysis plan (SAP)
X Non-commercial trial registry record (e.g. ClinicalTrials.gov record)
☐ Company-owned trial registry record (e.g. GSK Clinical Study Register record)
☐ “Grey literature” (e.g. unpublished thesis)
☐ Conference abstract(s) about the trial
☐ Regulatory document (e.g. Clinical Study Report, Drug Approval Package)
☐ Research ethics application
☐ Grant database summary (e.g. NIH RePORTER or Research Councils UK Gateway to Research)
☐ Personal communication with trialist
☐ Personal communication with the sponsor

Risk of bias assessment

Responses underlined in green are potential markers for low risk of bias, and responses in red are potential markers for a risk of bias. Where questions relate only to sign posts to other questions, no formatting is used.

Domain 1: Risk of bias arising from the randomization process

Signalling questions	Comments	Response options
**1.1 Was the allocation sequence random?**		Y / PY / PN / N / NI
**1.2 Was the allocation sequence concealed until participants were enrolled and assigned to interventions?**		Y / PY / PN / N / NI
**1.3 Did baseline differences between intervention groups suggest a problem with the randomization process?**		Y / PY / PN / N / NI
**Risk-of-bias judgement**		**Low** / High / Some concerns
Optional: What is the predicted direction of bias arising from the randomization process?		NA / Favours experimental / Favours comparator / Towards null /Away from null / Unpredictable

Domain 2: Risk of bias due to deviations from the intended interventions (*effect of assignment to intervention*)

Signalling questions	Comments	Response options
**2.1. Were participants aware of their assigned intervention during the trial?**	The medical student could be called if there were questions. It is not clarified if he/she was called and how this might have influenced unblinding and decision making.	Y / PY / PN / **N** / NI
**2.2. Were carers and people delivering the interventions aware of participants' assigned intervention during the trial?**		Y / PY / **PN** / N / NI
**2.3. If Y/PY/NI to 2.1 or 2.2: Were there deviations from the intended intervention that arose because of the trial context?**		**NA** / Y / PY / PN / N / NI
**2.4 If Y/PY to 2.3: Were these deviations likely to have affected the outcome?**		**NA** / Y / PY / PN / N / NI
**2.5. If Y/PY/NI to 2.4: Were these deviations from intended intervention balanced between groups?**		**NA** / Y / PY / PN / N / NI
**2.6 Was an appropriate analysis used to estimate the effect of assignment to intervention?**		**Y** / **PY** / PN / N / NI
**2.7 If N/PN/NI to 2.6: Was there potential for a substantial impact (on the result) of the failure to analyse participants in the group to which they were randomized?**		**NA** / Y / PY / PN / N / NI
**Risk-of-bias judgement**		**Low** / High / Some concerns
Optional: What is the predicted direction of bias due to deviations from intended interventions?		NA / Favours experimental / Favours comparator / Towards null /Away from null / Unpredictable

Domain 2: Risk of bias due to deviations from the intended interventions (*effect of adhering to intervention*)

Signalling questions	Comments	Response options
**2.1. Were participants aware of their assigned intervention during the trial?**		Y / PY / PN / **N** / NI
**2.2. Were carers and people delivering the interventions aware of participants' assigned intervention during the trial?**		Y / PY / **PN** /N / NI
**2.3. [If applicable:] If Y/PY/NI to 2.1 or 2.2: Were important non-protocol interventions balanced across intervention groups?**		NA / **Y** / PY / PN / N / NI
**2.4. [If applicable:] Were there failures in implementing the intervention that could have affected the outcome?**		NA / Y / PY / PN / **N** / NI
**2.5. [If applicable:] Was there non-adherence to the assigned intervention regimen that could have affected participants' outcomes?**		NA / Y / PY / PN / **N** / NI
**2.6. If N/PN/NI to 2.3, or Y/PY/NI to 2.4 or 2.5: Was an appropriate analysis used to estimate the effect of adhering to the intervention?**		**NA** / Y / PY / PN / N / NI
**Risk-of-bias judgement**		**Low** / High / Some concerns
Optional: What is the predicted direction of bias due to deviations from intended interventions?		NA / Favours experimental / Favours comparator / Towards null /Away from null / Unpredictable

Domain 3: Missing outcome data

Signalling questions	Comments	Response options
**3.1 Were data for this outcome available for all, or nearly all, participants randomized?**		Y / PY / PN / N / NI
**3.2 If N/PN/NI to 3.1: Is there evidence that the result was not biased by missing outcome data?**		NA / Y / PY / PN / N
**3.3 If N/PN to 3.2: Could missingness in the outcome depend on its true value?**		NA / Y / PY /PN / N / NI
**3.4 If Y/PY/NI to 3.3: Is it likely that missingness in the outcome depended on its true value?**		NA / Y / PY / PN / N / NI
**Risk-of-bias judgement**		Low / High / Some concerns
Optional: What is the predicted direction of bias due to missing outcome data?		NA / Favours experimental / Favours comparator / Towards null /Away from null / Unpredictable

Domain 4: Risk of bias in measurement of the outcome

Signalling questions	Comments	Response options
**4.1 Was the method of measuring the outcome inappropriate?**		Y / PY / PN / **N** / NI
**4.2 Could measurement or ascertainment of the outcome have differed between intervention groups?**		Y / PY / PN / **N** / NI
**4.3 If N/PN/NI to 4.1 and 4.2: Were outcome assessors aware of the intervention received by study participants?**		NA / **Y** / PY / PN / N / NI
**4.4 If Y/PY/NI to 4.3: Could assessment of the outcome have been influenced by knowledge of intervention received?**		NA / Y / PY / **PN** / N / NI
**4.5 If Y/PY/NI to 4.4: Is it likely that assessment of the outcome was influenced by knowledge of intervention received?**		NA / Y / PY / **PN** / N / NI
**Risk-of-bias judgement**		Low / High / **Some concerns**
Optional: What is the predicted direction of bias in measurement of the outcome?		NA / Favours experimental / Favours comparator / Towards null /Away from null / Unpredictable

Domain 5: Risk of bias in selection of the reported result

Signalling questions	Comments	Response options
**5.1 Were the data that produced this result analysed in accordance with a pre-specified analysis plan that was finalized before unblinded outcome data were available for analysis?**		Y / PY / **PN** / N / NI
**Is the numerical result being assessed likely to have been selected, on the basis of the results, from...**		
**5.2. ... multiple eligible outcome measurements (e.g. scales, definitions, time points) within the outcome domain?**		Y / PY / **PN** / N / NI
**5.3 ... multiple eligible analyses of the data?**		Y / PY / **PN** / N / NI
**Risk-of-bias judgement**		Low / High / **Some concerns**
Optional: What is the predicted direction of bias due to selection of the reported result?		NA / Favours experimental / Favours comparator / Towards null /Away from null / Unpredictable

Overall risk of bias

**Risk-of-bias judgement**		Low / High / **Some concerns**
Optional: What is the overall predicted direction of bias for this outcome?		NA / Favours experimental / Favours comparator / Towards null /Away from null / Unpredictable

Study details

Reference

Targeted HIV Screening in Eight Emergency Departments: The DICI-VIH Cluster-Randomized Two-Period Crossover Trial(2)

Study design

☐ Individually-randomized parallel-group trial

x Cluster-randomized parallel-group trial

☐ Individually randomized cross-over (or other matched) trial

For the purposes of this assessment, the interventions being compared are defined as

Experimental:	nurse-driven targeted HIV screening
Comparator:	diagnostic testing by physicians following usual care

**Specify which outcome is being assessed for risk of bias**	The primary outcome was the proportion of new diagnoses among included patients, which further refers to effectiveness
**Specify the numerical result being assessed.** In case of multiple alternative analyses being presented, specify the numeric result (e.g. RR = 1.52 (95% CI 0.83 to 2.77) and/or a reference (e.g. to a table, figure or paragraph) that uniquely defines the result being assessed.	The numeric result is presented in figure 2, the study flow diagram. It shows numbers of new HIV diagnoses per patients included in the respective arm, translated into n per 10,000. Resulting in 6 per 74,166 (control) versus 22 per 74,161 (intervention); translated into 0.8 versus 3.0 per 10,000.<br>The difference in identified proportion of new HIV diagnoses of the intervention versus control is expressed in a relative risk (RR 3.7; 95% CI 1.4 to 9.8).

Third numerical result is the additional new diagnoses during the intervention period (n = 16), representing 2.2 additional new HIV diagnoses per10,000 included patients (95% CI 1.3 to 3.6).

Is the review team's aim for this result…?

x to assess the effect of *assignment to intervention* (the 'intention-to-treat' effect)

☐ to assess the effect of *adhering to intervention* (the 'per-protocol' effect)

If the aim is to assess the effect of *adhering to intervention*, select the deviations from intended intervention that should be addressed (at least one must be checked):

X occurrence of non-protocol interventions

☐ failures in implementing the intervention that could have affected the outcome

☐ non-adherence to their assigned intervention by trial participants

Which of the following sources were obtained to help inform the risk-of-bias assessment? (tick as many as apply)

X Journal article(s) with results of the trial

☐ Trial protocol

☐ Statistical analysis plan (SAP)

X Non-commercial trial registry record (e.g. ClinicalTrials.gov record)

☐ Company-owned trial registry record (e.g. GSK Clinical Study Register record)

☐ "Grey literature" (e.g. unpublished thesis)

☐ Conference abstract(s) about the trial

☐ Regulatory document (e.g. Clinical Study Report, Drug Approval Package)

☐ Research ethics application

☐ Grant database summary (e.g. NIH RePORTER or Research Councils UK Gateway to Research)

☐ Personal communication with trialist

☐ Personal communication with the sponsor

Domain 1: Risk of bias arising from the randomization process

Signalling questions	Comments	Response options
**1.1 Was the allocation sequence random?**		**Y /** PY / PN / N / NI
**1.2 Was the allocation sequence concealed until participants were enrolled and assigned to interventions?**		**Y /** PY / PN / N / NI
**1.3 Did baseline differences between intervention groups suggest a problem with the randomization process?**		Y / PY / PN **/ N** / NI
**Risk-of-bias judgement**		**Low** / High / Some concerns
Optional: What is the predicted direction of bias arising from the randomization process?		NA / Favours experimental / Favours comparator / Towards null /Away from null / Unpredictable

Domain 2: Risk of bias due to deviations from the intended interventions (*effect of assignment to intervention*)

Signalling questions	Comments	Response options
**2.1. Were participants aware of their assigned intervention during the trial?**		Y / PY / PN / **N** / NI
**2.2. Were carers and people delivering the interventions aware of participants' assigned intervention during the trial?**		Y / PY / **PN** / N / NI
**2.3. If Y/PY/NI to 2.1 or 2.2: Were there deviations from the intended intervention that arose because of the trial context?**		NA / Y / PY / PN / N / NI
**2.4 If Y/PY to 2.3: Were these deviations likely to have affected the outcome?**		NA / Y / PY / PN / N / NI
**2.5. If Y/PY/NI to 2.4: Were these deviations from intended intervention balanced between groups?**		NA / Y / PY / PN / N / NI
**2.6 Was an appropriate analysis used to estimate the effect of assignment to intervention?**		**Y** / PY / PN / N / NI
**2.7 If N/PN/NI to 2.6: Was there potential for a substantial impact (on the result) of the failure to analyse participants in the group to which they were randomized?**		NA / Y / PY / PN / N / NI
**Risk-of-bias judgement**		**Low** / High / Some concerns
Optional: What is the predicted direction of bias due to deviations from intended interventions?		NA / Favours experimental / Favours comparator / Towards null /Away from null / Unpredictable

Domain 2: Risk of bias due to deviations from the intended interventions (*effect of adhering to intervention*)

**Signalling questions**	**Comments**	**Response options**
**2.1. Were participants aware of their assigned intervention during the trial?**		Y / PY / PN / **N** / NI
**2.2. Were carers and people delivering the interventions aware of participants' assigned intervention during the trial?**		Y / PY / **PN** /N / NI
**2.3. [If applicable:] If Y/PY/NI to 2.1 or 2.2: Were important non-protocol interventions balanced across intervention groups?**		NA / Y / PY / PN / N / NI
**2.4. [If applicable:] Were there failures in implementing the intervention that could have affected the outcome?**		NA / Y / PY / PN / **N** / NI
**2.5. [If applicable:] Was there non-adherence to the assigned intervention regimen that could have affected participants' outcomes?**		NA / Y / PY / PN / **N** / NI
**2.6. If N/PN/NI to 2.3, or Y/PY/NI to 2.4 or 2.5: Was an appropriate analysis used to estimate the effect of adhering to the intervention?**		NA / Y / PY / PN / N / NI
**Risk-of-bias judgement**		**Low** / High / Some concerns
Optional: What is the predicted direction of bias due to deviations from intended interventions?		NA / Favours experimental / Favours comparator / Towards null /Away from null / Unpredictable

Domain 3: Missing outcome data

Signalling questions	Comments	Response options
**3.1 Were data for this outcome available for all, or nearly all, participants randomized?**		**Y /** PY / PN / N / NI
**3.2 If N/PN/NI to 3.1: Is there evidence that the result was not biased by missing outcome data?**		NA / Y / PY / PN / N
**3.3 If N/PN to 3.2: Could missingness in the outcome depend on its true value?**		NA / Y / PY /PN / N / NI
**3.4 If Y/PY/NI to 3.3: Is it likely that missingness in the outcome depended on its true value?**		NA / Y / PY / PN / N / NI
**Risk-of-bias judgement**		**Low** / High / Some concerns
Optional: What is the predicted direction of bias due to missing outcome data?		NA / Favours experimental / Favours comparator / Towards null /Away from null / Unpredictable

Domain 4: Risk of bias in measurement of the outcome

**Signalling questions**	**Comments**	**Response options**
**4.1 Was the method of measuring the outcome inappropriate?**		Y / PY / PN / **N** / NI
**4.2 Could measurement or ascertainment of the outcome have differed between intervention groups?**		Y / PY / **PN** / N / NI
**4.3 If N/PN/NI to 4.1 and 4.2: Were outcome assessors aware of the intervention received by study participants?**		NA / Y / PY / PN / **N** / NI
**4.4 If Y/PY/NI to 4.3: Could assessment of the outcome have been influenced by knowledge of intervention received?**		NA / Y / PY / PN / N / NI
**4.5 If Y/PY/NI to 4.4: Is it likely that assessment of the outcome was influenced by knowledge of intervention received?**		NA / Y / PY / PN / N / NI
**Risk-of-bias judgement**		**Low** / High / Some concerns
Optional: What is the predicted direction of bias in measurement of the outcome?		NA / Favours experimental / Favours comparator / Towards null /Away from null / Unpredictable

Domain 5: Risk of bias in selection of the reported result

Signalling questions	Comments	Response options
**5.1 Were the data that produced this result analysed in accordance with a pre-specified analysis plan that was finalized before unblinded outcome data were available for analysis?**		Y / PY / PN **/ <u>N</u>** / NI
**Is the numerical result being assessed likely to have been selected, on the basis of the results, from...**		
**5.2. ... multiple eligible outcome measurements (e.g. scales, definitions, time points) within the outcome domain?**		Y / PY / **<u>PN</u>** / N / NI
**5.3 ... multiple eligible analyses of the data?**		Y / PY / **<u>PN</u>** / N / NI
**Risk-of-bias judgement**		**<u>Low</u>** / High / Some concerns
Optional: What is the predicted direction of bias due to selection of the reported result?		NA / Favours experimental / Favours comparator / Towards null /Away from null / Unpredictable

Overall risk of bias

**Risk-of-bias judgement**		**Low** / High / Some concerns
Optional: What is the overall predicted direction of bias for this outcome?		NA / Favours experimental / Favours comparator / Towards null /Away from null / Unpredictable
