# Supplementary material for: Systematic Review: Strategies for Improving HIV Testing and Detection Rates in European Hospitals
Source: Microorganisms. 2024 Jan 25;12(2):254. doi: 10.3390/microorganisms12020254 (PMC10892502; doi:10.3390/microorganisms12020254)
Supplement: Supplementary file 1 [file microorganisms-12-00254-s001.zip › microorganisms-2814635-supplementary/S5 ROBINS-I.pdf]

Supplement 5 (S5): Quality assessment using the Risk of Bias in Non-randomized Studies of Interventions (ROBINS-I) tool: traffic light and summary plot – HIV testing strategies in European hospitals

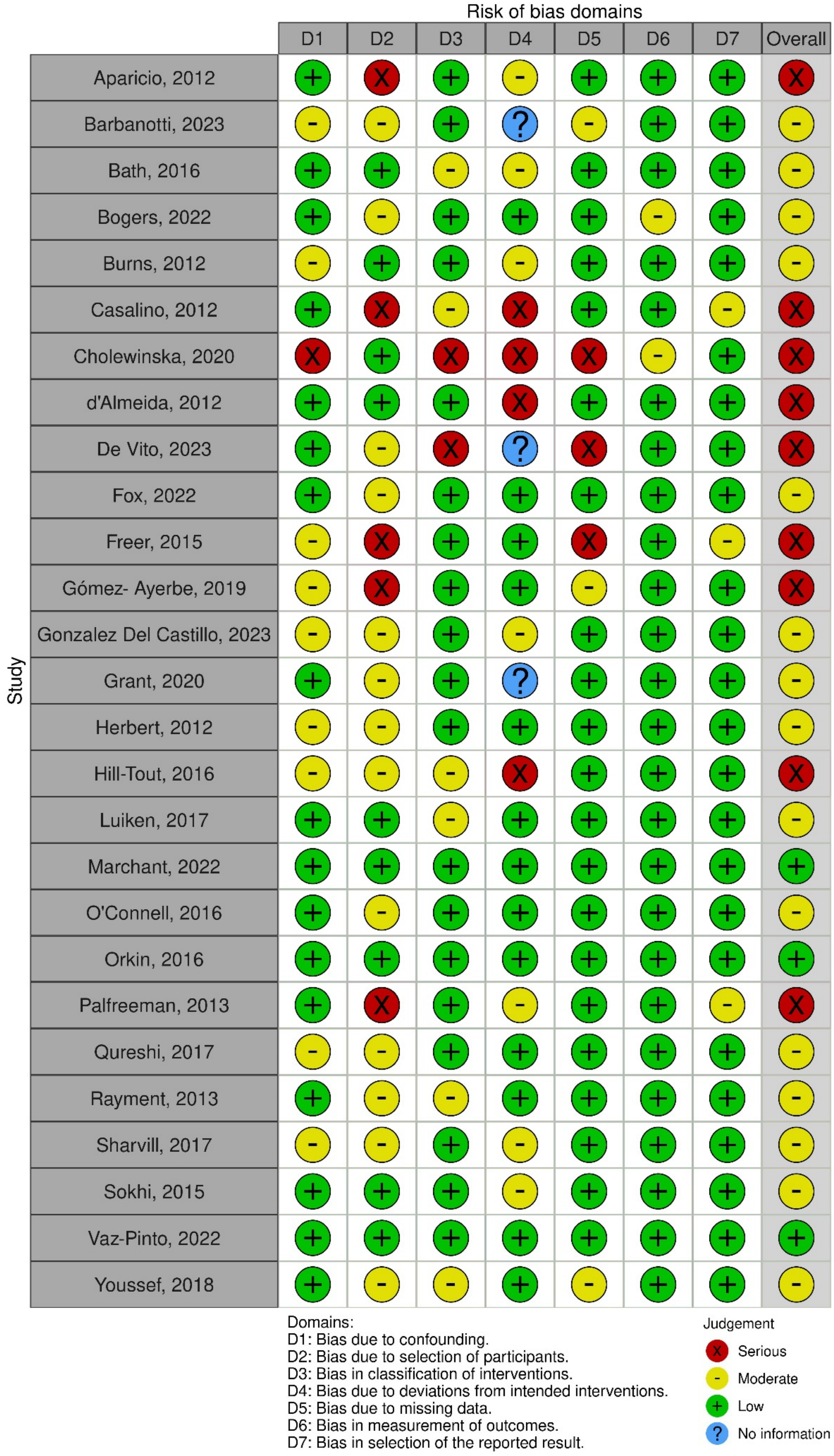

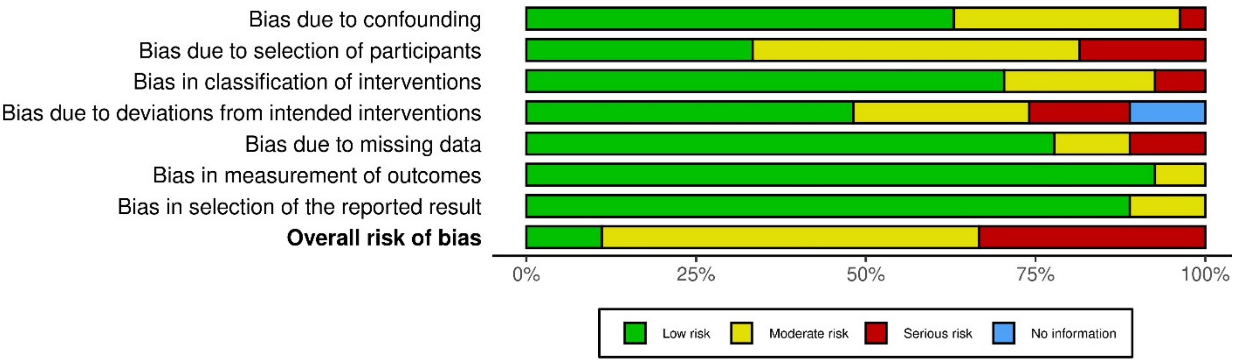

Tool: Sterne JAC, Hernán MA, Reeves BC, Savović J, Berkman ND, Viswanathan M, Henry D, Altman DG, Ansari MT, Boutron I, Carpenter JR, Chan AW, Churchill R, Deeks JJ, Hróbjartsson A, Kirkham J, Jüni P, Loke YK, Pigott TD, Ramsay CR, Regidor D, Rothstein HR, Sandhu L, Santaguida PL, Schünemann HJ, Shea B, Shrier I, Tugwell P, Turner L, Valentine JC, Waddington H, Waters E, Wells GA, Whiting PF, Higgins JPT. ROBINS-I: a tool for assessing risk of bias in non-randomized studies of interventions. BMJ 2016; 355; i4919; doi: 10.1136/bmj.i4919. Available from: https://www.riskofbias.info/welcome/home/current-version-of-robins-i/robins-i-tool-2016

Visualisation: Robvis. McGuinness, L.A.; Higgins, J.P.T. Risk-of-bias VISualization (robvis): An R package and Shiny web app for visualizing risk-of-bias assessments. Res Synth Methods 2021, 12, 55-61, doi:10.1002/jrsm.1411. Available from: https://www.riskofbias.info/welcome/robvis-visualization-tool
